# Effect of Preheating of Resin Luting Materials on Push-Out Bond Strength of Fiber Posts to Intraradicular Dentin

**DOI:** 10.3390/polym18040444

**Published:** 2026-02-10

**Authors:** Burcu Dikici, Nazlı Şirinsükan, Emre Alp Tüzüner, Esra Can

**Affiliations:** Department of Restorative Dentistry, Faculty of Dentistry, Yeditepe University, 34728 Istanbul, Turkey; nazli.sirinsukan@yeditepe.edu.tr (N.Ş.); emrealp.tuzuner@std.yeditepe.edu.tr (E.A.T.); esra.cansay@yeditepe.edu.tr (E.C.)

**Keywords:** preheating, fiber-reinforced flowable composite, injectable composites, push-out bond strength, fiber post

## Abstract

This study aimed to evaluate the effect of preheating on the push-out bond strength (PBS) and microhardness (HV) of fiber-reinforced flowable and injectable composites and to compare them with dual-cure resin–cement for post cementation. Fifty premolars were endodontically treated, and post spaces were prepared. Specimens were divided into five groups (*n* = 10) based on the resin luting material. After adhesive application, fiber posts were luted with dual-cure resin–cement (LinkForce), fiber-reinforced flowable composites (EverX Flow; non-heated/preheated), and injectable composites (G-aenial Universal Injectable; non-heated/preheated). After 24 h, roots were sectioned (coronal, middle, apical) for PBS testing (Instron). For HV, 10 specimens per resin luting material were prepared, and top/bottom microhardness was measured to assess the depth of cure. Data were analyzed with two-way ANOVA and post hoc Tukey tests (*p* < 0.05). Both types of resin luting material and preheating significantly affected PBS and HV (*p* = 0.0001). Preheated EverX Flow showed significantly higher PBS and HV than LinkForce, while G-aenial Injectable exhibited the lowest values (*p* < 0.05). Within each resin luting material, PBS significantly decreased from the coronal to the apical region (*p* = 0.0001). Preheated fiber-reinforced flowable composites demonstrate improved microhardness and adhesion, offering a reliable alternative to the dual-cure resin–cements for fiber post cementation.

## 1. Introduction

Endodontically treated teeth usually demonstrate extensive disruption of the dental hard tissues. When the remaining coronal structure is insufficient to support adequate retention for restorative treatment, intracanal posts can be used [1]. Among available post systems, fiber posts have gained wide acceptance due to their superior esthetics, mechanical properties, and elastic modulus, which is very close to dentin [2]. Unlike metallic posts, fiber posts transmit stresses more uniformly along the root and reduce the risk of catastrophic failure; however, debonding is the most common failure [3,4]. This failure is frequently associated with challenges in achieving adequate adhesion to root dentin, a mismatch between the prepared root canal and the post’s design or diameter [5]. Debonding can be observed at the dentin–resin–cement, composite core–fiber post, or fiber post–resin–cement interfaces [6,7,8]. Improving the adhesion between fiber posts, resin–cement, and root dentin enhances load transmission from the crown to the root, thereby improving the restoration’s durability [9].

Conventionally, dual-polymerizing resin–cements are used as luting materials for fiber posts to ensure adequate monomer conversion in the narrow, deep root canal environment, where the photoinitiators have reduced irradiance [10]. The use of translucent fiber posts allows deeper light penetration into the root canal [11], thereby improving resin–cement polymerization; however, inadequate polymerization in the apical regions of the root canal has still been observed [12]. Incomplete polymerization can compromise the mechanical properties of the resin–cement, resulting in poor adhesion and clinical performance [13]. In addition, dual-cure resin–cements exhibit limited load-bearing capacity due to their lower filler content, which may lead to microfractures under functional stress [8,14]. Therefore, light-cure resin composites were considered as an alternative to dual-cure resin–cements to enhance the structural durability of fiber post restorations. Recent studies have shown improved adhesion of fiber posts to irregular root canals, higher push-out bond strength to root dentin (PBS), and a more favorable stress distribution with light-cure resin composites compared with dual-cure resin–cements [8,15,16]. Bulk-fill resin composites, in particular, exhibit efficient light transmission through the post and an increased degree of conversion, resulting in superior mechanical properties compared with dual-cure resin–cements [17]. Although these materials enable deeper curing than conventional resin composites, concerns remain regarding their depth of cure, microhardness, and polymerization stress. To enhance these properties, preheating techniques have been introduced to increase resin flowability, improve adaptation, and raise the degree of conversion [18]. By achieving a higher depth of cure and reducing polymerization stress, these materials may provide a more reliable interface and reduce adhesive failures [19].

Although numerous studies have investigated the mechanical properties and bonding behavior of fiber posts and various resin luting materials, limited information is available on the effects of preheating techniques of resin composites on fiber post luting. The polymerization behavior, depth of cure, and bonding performance of these materials inside the root canal remain insufficiently explored. Therefore, this study aims to evaluate the preheating effect on PBS and the HV of fiber-reinforced flowable composite, and injectable composites and, compare them with dual-cure resin–cement for fiber post cementation.

The tested null hypotheses are as follows:

(1) There is no significant difference in the PBS of fiber posts luted with dual-cure resin–cement, preheated and non-heated fiber-reinforced flowable composite, and preheated and non-heated injectable composite.
(2) There is no significant difference in the PBS of fiber posts between the coronal, middle, and apical thirds of the root canal dentin.
(3) There is no significant difference in the microhardness between dual-cure resin–cement, preheated and non-heated fiber-reinforced flowable composite, and preheated and non-heated injectable composite.
(4) There is no significant difference in the microhardness of the same resin luting materials at different depths.

## 2. Materials and Methods

The materials used in the study are listed in Table 1.

This research received approval from the Research Ethics Committee of Yeditepe University (612). Power analysis was performed using the G-POWER software (G*Power 3.1 Software; Düsseldorf, Germany), assuming an effect size of 0.5, 85% power, and a 0.05 margin of error, to determine the sample size required. Based on this analysis, 10 tooth specimens were allocated to each group for both the PBS and microhardness tests.

### 2.1. PBS Test

A total of fifty intact, single-rooted human premolars with a minimum root length of 17 mm were utilized. Tooth selection was carried out according to specific criteria: complete apexification, extracted for surgical, prosthetic, orthodontic, or periodontal reasons and caries-free. The selected teeth were randomly allocated into experimental groups using a computer-generated randomization sequence, and all subsequent procedures and data analyses were performed by a blinded operator to minimize bias. Following extraction, soft tissues covering the root surfaces were removed via ultrasonic scaling, and the teeth were stored in 0.5% chloramine T solution at room temperature until use. All teeth were used within 6 months of extraction. The coronal portions were removed 2 mm above the cement–enamel junction (CEJ) using a water-cooled, low-speed diamond saw (IsoMet 1000, Buehler Ltd., Lake Bluff, IL, USA). This preparation design was chosen to mimic clinical cases where extensive structural loss (ferrule ≤ 2 mm) justifies the use of a fiber post over more conservative options, such as endocrowns [20,21]. Root canal treatment was performed on all teeth.

Working lengths were established 1.0 mm short of the apex. Biomechanical preparation was conducted with ProTaper Next rotary instruments (Dentsply Sirona, Charlotte, NC, USA) up to size X5 (50/0.06 taper). Canals were irrigated with a 1% sodium hypochlorite (NaOCl) solution following each file change. The final disinfection sequence included 3 mL of 1% NaOCl, 3 mL of 17% EDTA for 60 s, and a final 1 min rinse with 1% NaOCl, followed by 10 mL of distilled water. Once dried with paper points, the canals were filled with AH Plus sealer and gutta-percha (Dentsply Sirona, NC, USA) via the cold lateral compaction technique. Cavity accesses were temporarily filled (G-aenial Achord, GC), and specimens were incubated at 37 °C for one week.

Post spaces were created using size 2 Easy-Post-Precision Drills (Dentsply Maillefer, Tulsa, OK, USA), ensuring a 5 mm apical gutta-percha seal remained. Drill dimensions (1.5 mm coronal/0.8 mm apical) and post dimensions (1.2 mm coronal/0.5 mm apical) were selected to provide a uniform cement film thickness of approximately 0.3 mm. Standard fiber posts (φ 1.2 mm; GC) were used for all specimens to ensure uniformity in fit and retention following post-space preparation. All teeth received the same adhesive treatment protocol before post-cementation. A universal adhesive system (G-Premio BOND, GC) mixed 1:1 with Dual Cure Activator (DCA, GC) was applied to the post space using a microbrush for 20 s, gently air-dried for 5 s, and excess adhesive was removed with paper points according to the manufacturer’s instructions and not polymerized.

Before cementation, all fiber posts were cleaned with alcohol and coated with G-Multi Primer (GC Europe, Leuven, Belgium) using a microbrush for 60 s and air-dried. Five different cementation protocols were evaluated according to the type of resin luting material used: dual-cure resin–cement (G-CEM LinkForce, GC), fiber-reinforced flowable composite (EverX Flow, GC), preheated fiber-reinforced flowable composite (EverX Flow, GC; preheated to 55 °C before the application), injectable composite resin (G-aenial Universal Injectable, GC) and preheated injectable composite resin (G-aenial Universal Injectable, GC, preheated to 55 °C before the application). LinkForce was used as a control group. The respective resin luting materials were injected into the post spaces using an automix tip and applied onto the post surfaces. Fiber posts were then seated into the root canals under finger pressure, and excess material was carefully removed.

In all groups, light-curing was performed for 80 s from the coronal direction using an LED curing unit (Bluephase N, Ivoclar Vivadent, Schaan, Liechtenstein). The specimens were then left undisturbed for 4 min to ensure complete polymerization. After storage in distilled water at 37 °C for 24 h, all specimens were embedded in autopolymerizing acrylic resin (SC; Imicryl, Konya, Turkey) to facilitate sectioning and handling during testing. This protocol was chosen to ensure that the initial bonding and polymerization of the resin luting materials were completed under controlled conditions, avoiding any potential thermal interference from the exothermic reaction of the acrylic resin. A low speed, water cooled diamond saw (Isomet 1000; Buehler, Lake Forest, IL, USA) was used for sectioning, starting from the coronal third of the root and proceeding apically. Each root was sectioned perpendicular to its long axis to obtain three 1 mm thick slices, corresponding to the coronal, middle, and apical thirds of the post space. PBS testing was then performed separately for each section using a universal testing machine (Instron Model 3345, Instron Corp., Norwood, MA, USA) at a crosshead speed of 0.5 mm/min until post-dislodgement occurred. The maximum load at failure (N) was recorded for each section and converted to bond strength (MPa) by dividing by the bonded surface area. Following the PBS test, bonded surfaces of the dislodged specimens were examined under a scanning electron microscope (SEM; 6335-F, JEOL Ltd., Tokyo, Japan) to evaluate the interfacial morphology between the post, resin–cement/composite, and dentin.

### 2.2. Microhardness Tests for Depth of Cure Evaluation

To assess the depth of cure for each resin composite, the microhardness of the composite was measured at various thicknesses (2, 3, 4, and 6 mm). Black cylindrical stainless-steel molds with a 2 mm diameter and 10 mm thickness were used to prevent light reflection. A total of 50 specimens (10 from each material) were prepared from dual-cure resin–cement, fiber-reinforced flowable composite, preheated fiber-reinforced flowable composite, G-aenial Injectable, and preheated G-aenial Injectable for measuring surface microhardness profiles. The materials were put into a metallic circular mold, sealed with a transparent Mylar band, compressed using 1 mm thick microscopic glass slides on opposing sides, and polymerized (40 s). All specimens were preserved in a dry incubator for 24 h before measurement. A Vickers microhardness indenter (Buehler Ltd., Lake Bluff, IL, USA) was employed for the microhardness test, utilizing a 50 g load for 15 s. Three indentations were created at each millimeter, and the average microhardness was assessed and documented. The sufficient depth of cure for each specimen was determined as the minimum depth at which the microhardness value of the sample surface exceeded 80% of the top surface value. Measurements were performed according to the ISO 4049 procedure [22]. Any unpolymerized, soft lower section was excised using a spatula.

### 2.3. SEM Analyses and Failure Type Evaluation

To evaluate the interfacial morphology, representative samples from each experimental group were preserved in a 10% neutral buffered formalin solution for a 24 h period. Following fixation, the specimens were mounted on metallic stubs and allowed to undergo desiccation at room temperature. To ensure electrical conductivity for high-resolution imaging, gold sputter-coating was applied. The microstructural features of the bonded interfaces were then analyzed with a scanning electron microscope (SEM; 6335-F, JEOL Ltd.).

To identify the failure patterns, the samples were examined under a stereomicroscope (Carl Zeiss Inc., Oberkochen, Germany) at 40× magnification. The failure modes were categorized into five types based on the fractured interface:

Type I: Adhesive Failure at the interface between the resin luting material and the intraradicular dentin.

Type II: Adhesive Failure between the fiber post and the resin luting material, leaving the post surface essentially clean.

Type III: Cohesive Failure within the resin luting material.

Type IV: Cohesive Failure within the fiber post structure.

Type V: Mixed Failure, a combination of two or more of the failure patterns, involving both adhesive and cohesive elements [23].

### 2.4. Statistical Analysis

Statistical analyses were carried out using NCSS (Number Cruncher Statistical System) 2007 Statistical Software (Kaysville, UT, USA). Descriptive statistics, including mean and standard deviation, were used to summarize the quantitative variables. Before conducting inferential testing, the distributional properties of all continuous variables were assessed using the Shapiro–Wilk normality test to ascertain the suitability of parametric approaches.

For normally distributed variables, comparisons between the various composite groups and root regions were conducted using two-way analysis of variance (ANOVA). Following the identification of significant main or interaction effects, post hoc pairwise comparisons were conducted using the Tukey multiple comparison test to explain specific group differences.

A repeated-measures two-way ANOVA was used to assess variations in microhardness values at different depths within the same composite resin materials. Substantial differences were further analyzed using Tukey post hoc testing to ascertain the differences between measurement levels.

The relationship between PBS values and microhardness measurements at various depths was assessed using Pearson correlation analysis. Correlation coefficients (r) and their associated *p*-values were presented to identify the size and quality of the relationships examined. All statistical tests were two-tailed, and the level of significance was set at *p* < 0.05.

## 3. Results

### 3.1. PBS Results

**Figure 1:**
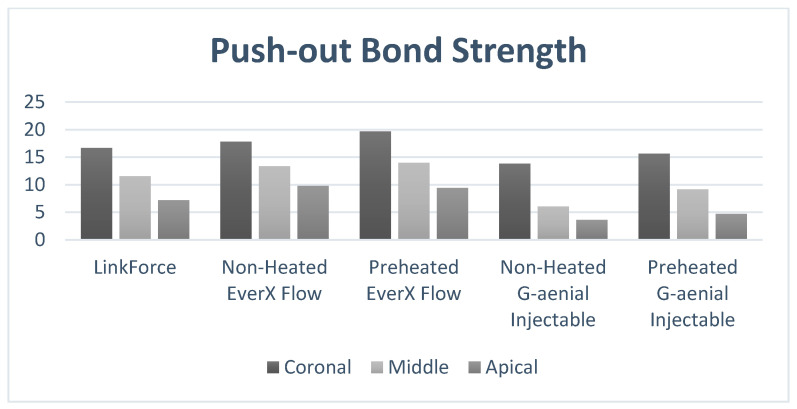
PBS (MPa) of fiber posts at the coronal, middle, and apical regions.

The mean and standard deviations of the PBS in coronal, middle, and apical regions of the root are summarized in Table 2 and Figure 1. Both the type of resin luting material and the root region significantly influenced the PBS (*p* = 0.0001) (Table 3 and Table 4).

In every region, non-heated G-aenial Injectable exhibited a statistically significant lower PBS than the other tested materials, while preheated EverX Flow showed the highest (Table 2).

Within each resin luting material group, PBS significantly decreased from the coronal to the apical region (*p* = 0.0001) (Table 2). Pairwise comparisons indicated that the apical region values were significantly lower than both the coronal and middle values, while the middle region values were also significantly lower than those in the coronal region (*p* = 0.0001) (Table 3).

Preheated EverX Flow yielded the highest bond strength for all regions (coronal: 19.69 ± 1.57 MPa; middle: 13.99 ± 1.93 MPa; apical: 9.43 ± 1.12 MPa) (Table 2). Preheated and non-heated G-aenial Injectable composites demonstrated a statistically significant decrease in bond strength from the coronal to the apical region (Table 2 and Table 3). The coronal region’s values were significantly higher than those in the middle and apical thirds, and middle values were likewise significantly higher than apical values (*p* = 0.0001) (Table 3).

In the coronal third, preheated EverX Flow demonstrated the highest PBS among all resin luting materials. Non-heated EverX Flow and LinkForce had comparable PBS values, which were significantly higher than those of the non-heated and preheated G-aenial Injectable composite. Preheated G-aenial Injectable exhibited comparable coronal PBS values with LinkForce, whereas non-heated G-aenial Injectable yielded the lowest coronal PBS values among all materials (*p* < 0.05) (Table 2).

In the middle third, preheated and non-heated EverX Flow demonstrated the highest PBS among all resin luting materials. Furthermore, EverX Flow and LinkForce exhibited statistically comparable PBS values (*p* > 0.05), and both have statistically significantly higher PBS values than preheated and non-heated G-aenial Injectable (*p* < 0.05). Non-heated G-aenial Injectable remained the weakest material, presenting the lowest middle PBS values among all groups (*p* < 0.05) (Table 2).

In the apical third, preheated and non-heated EverX Flow demonstrated the highest PBS values with no significant difference between them (*p* > 0.05). Preheated and non-heated G-aenial Injectable composites demonstrated the lowest PBS among all groups (*p* < 0.05). LinkForce showed statistically significantly lower PBS than preheated and non-heated EverX Flow, while yielding higher PBS than both preheated and non-heated G-aenial Injectable composite (Table 2).

### 3.2. Microhardness Test for Depth of Cure Evaluation

Statistically significant differences were observed between the different resin luting materials at each depth (*p* = 0.001), and the rate of microhardness reduction differed markedly between materials (Table 5 and Table 6). At the top surface and 2, 3, 4, and 6 mm depths, significantly preheated EverX Flow showed the highest microhardness values (*p* < 0.05), followed by LinkForce, which exhibited comparable microhardness values with non-heated EverX Flow (*p* > 0.05) (Table 5).

At the top surface, while there were no statistically significant differences between non-heated EverX Flow and LinkForce (*p* = 0.949) and preheated and non-heated G-aenial Injectable composites (*p* = 0.950), significant differences between the remaining groups were observed (*p* = 0.0001). At 2 mm, non-heated EverX Flow and LinkForce (*p* = 0.993) and preheated and non-heated G-aenial Injectable composites (*p* = 0.840) remained comparable, with significant differences between the other groups (*p* = 0.0001). At 3 mm, similar trends were observed: no significant differences between non-heated EverX Flow and LinkForce (*p* = 0.989) and preheated and non-heated G-aenial Injectable (*p* = 0.514), with significant differences between the remaining groups (*p* = 0.0001). At 4 and 6 mm, no significant difference was found between non-heated EverX Flow and LinkForce (*p* = 0.996, *p* = 0.852), whereas significant differences were observed between the remaining groups (*p* = 0.0001). At 6 mm, non-heated G-aenial Injectable did not polymerize adequately; therefore, surface microhardness values could not be obtained (Table 5).

A depth-dependent reduction in microhardness was evident for all materials, with the greatest decrease observed between the uppermost surface and deepest level (*p* < 0.05) (Table 5 and Table 6). At the top surface and 2, 3, 4, and 6 mm depths, preheated EverX Flow exhibited significantly higher HV values than all groups (*p* = 0.0001). At each corresponding depth, non-heated EverX Flow showed significantly higher HV than preheated and non-heated G-aenial Injectable (*p* = 0.0001) (Table 5).

For all materials, HV decreased progressively and significantly from the top surface to deeper levels (*p* < 0.05). A significant positive correlation was observed between HV and PBS across all regions (Table 7). In the coronal third, HV at the top surface and 2, 3, 4, and 6 mm depths positively correlated with PBS values (r = 0.756, 0.744, 0.753, 0.785, and 0.679, respectively; *p* = 0.0001). Similar positive correlations were observed in the middle third (r = 0.770, 0.759, 0.771, 0.824, 0.689, respectively; *p* = 0.0001), where the strongest correlations were observed. In the apical third, microhardness and PBS demonstrated significant positive correlations (r = 0.787, 0.774, 0.789, 0.818, 0.745, respectively; *p* = 0.0001). When all measurements were evaluated, the overall correlations remained statistically significant.

### 3.3. SEM Results

**Figure 2:**
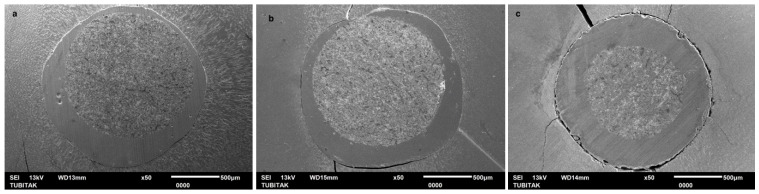
SEM images of adhesive/bonded interface of dual-cure resin–cement LinkForce: (**a**) coronal, (**b**) middle, and (**c**) Apical.

The SEM images of Linkforce group demonstrated a consistently uniform and adequate interface across all three regions (Figure 2).

**Figure 3:**
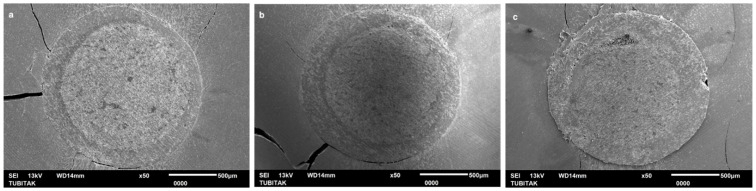
SEM images of adhesive/bonded interface of preheated EverX Flow: (**a**) coronal, (**b**) middle, and (**c**) apical.

The SEM micrographs revealed a well-integrated, homogeneous interface between the preheated EverX Flow and the fiber post across all three root regions (Figure 3). In the coronal, middle, and apical regions, an adequate adaptation was observed, correlating with the high bond strength values recorded. The preheating of EverX has markedly improved its flowability and conformity to the substrate, yielding a dense, void-free interfacial complex.

**Figure 4:**
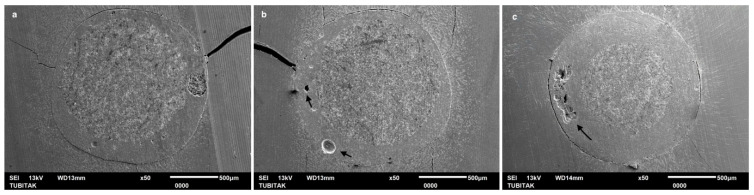
SEM images of adhesive/bonded interface of non-heated EverX Flow: (**a**) coronal, (**b**) middle, and (**c**) apical (Arrows show voids).

In contrast, while non-heated EverX Flow exhibited PBS values comparable to the preheated EverX Flow in some areas, the SEM evaluation identified critical structural deficiencies (Figure 4). Although a stable interface was present in the coronal region, the apical third revealed inadequately polymerized fibers (Figure 4). Furthermore, air bubbles were detected within the resin luting composite in the middle third.

**Figure 5:**
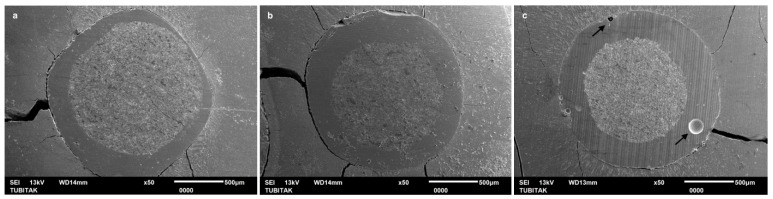
SEM images of adhesive/bonded interface of preheated G-aenial Injectable: (**a**) coronal, (**b**) middle, and (**c**) apical (Arrows show voids in the apical).

**Figure 6:**
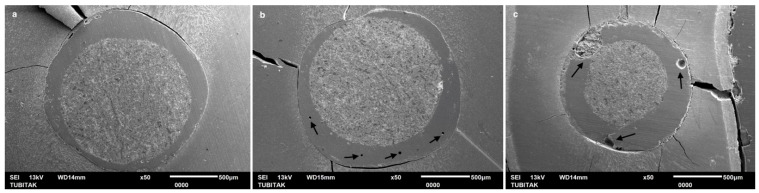
SEM images of adhesive/bonded interface of non-heated G-aenial Injectable: (**a**) coronal, (**b**) middle, and (**c**) apical (Arrows show voids in the middle and apical).

The preheated G-aenial Injectable showed a favorable interface in the coronal and middle sections; however, air bubbles were still present within the apical region (Figure 5, Arrows). Non-heated G-aenial Injectable group exhibited the most compromised microstructural integrity. While the coronal bond appeared adequate, a significant increase in void formation and unpolymerized areas was observed in the middle and apical regions (Figure 6, Arrows). These findings suggest that insufficient light attenuation at greater depths severely limits the polymerization of the injectable composite.

**Figure 7:**
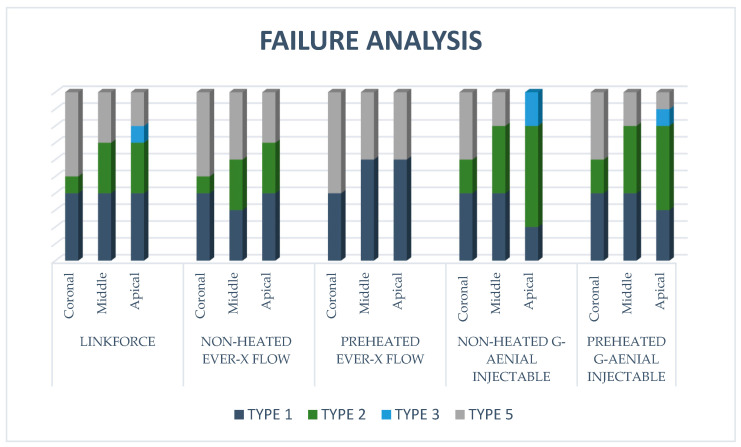
Failure analysis of resin luting materials in different dentin depth.

The distribution of failure modes by root canal region (coronal, middle, and apical) across all experimental groups is shown in Figure 7. A comprehensive quantitative analysis revealed that Type 1 (Adhesive Failure at the interface between the resin luting material and the intraradicular dentin) was the dominant failure pattern throughout the study, particularly in the apical and middle third. In the Preheated Ever-X Flow group, a distinct pattern was observed: only Type 1 and Type 5 (mixed failure) were recorded across all regions. Notably, Type 5 failures were significantly more frequent in the coronal regions of both the Preheated EverX Flow and Preheated G-aenial Injectable groups, suggesting that the reduction in viscosity through preheating facilitated an integrated bond in the coronal segments. Type 2 failure (Adhesive Failure between the fiber post and the resin luting material) was material-specific, occurring exclusively in the G-aenial Injectable groups (both non-heated and preheated). This failure mode was most prominent in the apical and middle regions of these groups, indicating a relative weakness in the interfacial bond between the fiber post and the injectable composite compared with the fiber-reinforced groups. Regarding cohesive failures, Type 3 (cohesive failure within the resin luting material) was rarely observed, identified only in the apical segments of the LinkForce and G-aenial Injectable groups. Notably, no Type 4 (cohesive failure of the post) was recorded in any experimental group, indicating that the structural integrity of the fiber-reinforced posts was maintained throughout the push-out testing.

## 4. Discussion

The main finding of this study was that preheating significantly enhances the push-out bond strength (PBS) of fiber-reinforced flowable composite and universal injectable composite, with results varying across root regions (Table 2). Compared to non-heated composites, preheated EverX Flow exhibited significantly higher PBS in the coronal region, whereas preheated G-aenial Injectable achieved significantly higher PBS in the coronal and middle sections. These findings suggest that while preheating is essential for optimizing interfacial bonding, its effectiveness is influenced by the depth of intraradicular dentin.

Regarding PBS in the coronal region, preheated EverX Flow achieved the highest bonding among all tested groups. Consistent with the PBS results, SEM analysis also demonstrated a highly integrated interfacial complex, characterized by a tight adaptation between the fiber post and the preheated EverX Flow (Figure 3). On the other hand, while non-heated G-aenial Injectable composite yielded significantly the lowest bond strength, preheated G-aenial Injectable had a comparable PBS result with LinkForce. These findings indicate that preheating of fiber-reinforced flowable composite and universal injectable composite used as luting materials for fiber posts significantly enhances PBS in the coronal region of intraradicular dentin. The benefits of preheating, such as reduced viscosity, improved flow, and increased monomer mobility, may enhance coronal adaptation, in which access to light and material flow is more favorable [24,25]. When preheated and non-heated EverX Flow were compared, the PBS results showed that although preheating improved the coronal bond strength, both approaches exhibited comparable results in the middle and apical regions of intraradicular dentin. However, despite the statistically comparable PBS values, SEM evaluation revealed critical structural defects in non-heated EverX Flow (Figure 4), including air bubbles in the middle region and incomplete polymerization in the apical region (Figure 4b,c), while preheated EverX Flow demonstrated superior interfacial integrity (Figure 3). These microscopic deficiencies suggest that, even when initial bond strengths appear similar, the internal structural compromise in the non-heated group may negatively influence the long-term clinical durability and the overall stability of the post-core complex under functional loading. Moreover, unpolymerized monomers may also induce biocompatibility concerns due to the cytotoxic effect, potentially leading to persistent surrounding tissue irritation and chronic inflammatory responses [26].

Linkforce showed statistically similar performance to non-heated EverX Flow in the coronal and middle regions; however, in the apical region, it yielded lower PBS than both EverX Flow groups. On the other hand, Linkforce resulted in significantly higher bonding performance than both G-aenial injectable groups in the middle and apical regions. SEM evaluation corroborated these findings, revealing a remarkably uniform interface in the Linkforce group (Figure 2), whereas both the preheated and non-heated G-aenial Injectable displayed prominent voids, particularly localized in the apical region (Figure 5 and Figure 6). These findings indicate that, although LinkForce benefits from dual-cure polymerization, which enhances interfacial integrity in deeper regions compared to G-aenial injectable groups, its adhesive performance remains inferior to that of both EverX Flow groups form in deeper regions, where light attenuation and reduced monomer mobility become limiting factors [27]. According to Üçtaşlı et al.’s findings [28], utilizing flowable fiber-reinforced composite as a resin luting material effectively eliminates the “weak link” typically found at the post–dentin interface by creating a chemically and mechanically integrated fiber-to-fiber complex. The random orientation of the short fibers within the flowable fiber-reinforced composite matrix redistributes functional stresses and arrests crack propagation, thereby enhancing the overall load-bearing capacity of the restoration compared with conventional resin–cements [28,29,30]. The use of a translucent fiber post may have further improved light transmission, allowing enhanced light penetration and thus deeper cure, although light intensity diminishes in the apical regions [31]. The fiber post serves as an effective light-transmitting rod, while the translucent fiber-reinforced flowable composite facilitates light transmission over extended distances [32,33]. The simultaneous application of a prefabricated fiber post and translucent fibers improves polymerization of the resin–cement in the deep apical area, resulting in higher bond strength [30,34]. These findings align with those of Suni et al. [8], who attributed this enhanced performance to the structural integrity and the resulting micro-mechanical interlocking between the protruded fibers of the resin luting material and the fiber post surface. They highlighted that the random orientation of fibers in short-fiber-reinforced composites facilitates light scattering and improves polymerization depth. This study adds a critical dimension by demonstrating that preheating appears to maximize the benefits of the semi-interpenetrating polymer network structure.

The lower bond strength of LinkForce compared to EverX Flow can also be attributed to its lower filler content, which leads to higher polymerization shrinkage and increased stress at the adhesive interface [35,36]. EverX Flow’s higher filler loading (70% by weight) effectively mitigates polymerization stresses, enhancing interfacial adhesion in the high-C-factor root canal environment [37]. Consequently, while LinkForce relies on its dual-cure chemistry, the higher filler–matrix ratio and translucency of EverX Flow may have provided a more potentially facilitating depth of cure and microhardness, as well as increased resistance to debonding [38]. Previous studies by Lassila et al. [29] and Frater et al. [30] have shown that a fiber-reinforced composite material can be effectively polymerized within the root canal adjacent to a specific fiber post, achieving microhardness levels comparable to those of dual-cure materials. These researchers reported that both dual-cure resin–cements and fiber-reinforced composites could maintain sufficient polymerization to a depth of 8 mm. Their findings validate the use of flowable fiber-reinforced composites as a viable alternative to dual-cure cements for the adhesion of the fiber posts to dentin. Based on the results of this study, both the type of the resin luting material and the preheating play a critical role in the PBS of fiber posts to intraradicular dentin; therefore, the first null hypothesis—that there is no significant differences in the PBS of fiber posts luted with dual-cure resin–cement, preheated and non-heated fiber-reinforced flowable, and injectable composite—was rejected.

The analysis of failure modes in the present study revealed that Type 1 (adhesive failure between resin luting material and intraradicular dentin) was the most prevalent pattern across all experimental groups. This finding underscores the inherent difficulty of the adhesive procedure in the long, narrow geometry of the root canal, where uniform application of the adhesive system and effective light-curing are technically challenging. The higher prevalence of adhesive failures also confirms that the mechanical forces during the PBS test were effectively concentrated at the adhesive interface, enabling a rigorous evaluation of bond strength. However, Type 5 (Mixed failure) was mostly observed in the coronal regions of the preheated groups. This can be attributed to the improved stress distribution achieved through preheating. According to Le Bell et al. [33] and Lassila et al. [29], when endodontic posts achieve an optimized bond, stresses are more effectively transferred to the surrounding substrates rather than being concentrated solely at the interface, leading to multiple failure modes. The reduction in viscosity through preheating likely facilitated more integrated interfacial adaptation, allowing the assembly to resist dislodgment more effectively. Interestingly, Type 3 (Cohesive failure in resin luting material) was rarely observed and was identified only in the apical part of the Linkforce and G-aenial Injectable groups. This limited occurrence is considered primarily material-dependent, suggesting that although the luting agents possessed internal strength, stress concentrations at the interfaces typically initiated debonding before cohesive fracture could develop within the material bulk. The exclusive occurrence of Type 2 (adhesive failure between the fiber post and resin luting material) in the G-aenial Injectable groups, and its absence in the preheated EverX Flow groups, can be attributed to the structural compatibility between the materials. Because EverX Flow is a short-fiber-reinforced composite, its structural similarity to the fiber post likely enhanced interfacial synergy. This “fiber-to-fiber” compatibility, potentially supported by a semi-interpenetrating polymer matrix, provides a more reliable chemical and mechanical bond than the particulate-filled G-aenial Injectable. As demonstrated by studies, stress concentrations at the cement-to-post interface are more likely to initiate debonding via brittle crack propagation when such structural and chemical harmony between the post and the luting agent is lacking [39,40].

Across all resin luting composite materials, PBS values followed a consistent regional pattern and significantly decreased from coronal to apical region (Table 3); therefore, the second hypothesis—that there are no significant differences in the PBS of fiber posts between the coronal, middle, and apical thirds of the root canal—was also rejected. A progressive decrease in bond strength from the coronal to the apical region was consistent with previous reports [17,41] and can be attributed to several factors, including insufficient polymerization linked to the decreased degree of monomer conversion in deeper areas, reduced light transmission, limited accessibility, and diminished dentin permeability [31,42,43]. These anatomical and optical constraints have amplified the performance differences between the tested materials. SEM images revealed air bubbles in the preheated and non-heated G-aenial injectable groups in the apical region (Figure 5 and Figure 6), indicating that the resin luting material did not achieve complete polymerization. Furthermore, the increased volume of resin–cement apically is due to the progressive taper of fiber posts. This is combined with the inherently challenging adhesion to apical radicular dentin—characterized by fewer, narrower, and less dense dentinal tubules—further compromises bonding effectiveness [44]. SEM analysis corroborated these findings, revealing that the resin luting material layer was thicker in the apical region than in the coronal region in all groups (Figure 2, Figure 3, Figure 4, Figure 5 and Figure 6). Similarly, Pinto et al. [45] attributed lower bond strength in the apical region to limited adhesive access, reduced light-curing energy penetration, and decreased dentin tubule density and diameter. In contrast, Shimizu et al. [46] reported comparable bond strengths across apical, middle, and coronal regions in bovine teeth when different adhesive systems were used, and they found no differences between the regions of root dentin.

In parallel with the PBS results, significant differences were observed among the resin luting materials regarding microhardness (HV), demonstrating a strong positive correlation between bonding performance and microhardness; thus, the third hypothesis was also rejected. The mechanical strength and surface hardness of resin materials are primarily determined by their filler content and the density of the polymer network [47]. The strength of the material increases with increasing filler loading [47]. With respect to HV, preheated EverX Flow, with a filler content of 70% by weight, consistently achieved the highest values across all depths (Table 5). In addition to filler load, this improvement can also be attributed to the increased thermal energy provided by preheating, which reduces the viscosity of the resin matrix and enhances the mobility of free radicals, leading to a higher degree of monomer conversion and a more stable polymer network [18,48]. This effect is reflected in the increased microhardness observed among preheated composite resins [49,50]. Consistently, Srikumar et al. showed that preheating (55 °C) of EverX Flow resulted in higher microhardness and depth of cure compared with Beautifil Bulk Restorative and G-aenial Posterior Composite [48]. In contrast, for G-aenial Injectable, the effect of preheating was more depth-dependent: while preheating significantly improved HV at 4 mm and 6 mm, no significant differences were observed at the top, 2 mm, and 3 mm depths compared to the non-heated group. Additionally, while non-heated EverX Flow (70 wt%) and LinkForce (63 wt%) exhibited comparable values, the G-aenial Injectable groups showed the lowest HV despite their 69 wt% filler loading, whereas preheated EverX Flow resulted in the highest HV across all depths, suggesting that both filler technology and preheating are more decisive for surface microhardness than weight percentage alone. Consistent with our results, Basheer et al. [51] also reported that G-aenial Universal Injectable showed significantly lower HV than other materials, further emphasizing that high-strength injectable materials may show varied mechanical performance due to the specific filler technologies and resin matrices used, which directly influence the material’s resistance to deformation [51].

Similar to the PBS result, all resin luting materials showed a progressive, statistically significant decrease in microhardness with increasing measurement depth, with significant differences observed between materials; thus, the fourth hypothesis was also rejected. Consistent with HV results, Rueggeberg et al. [52] also found higher HV on the top surface of composite specimens compared to the bottom. This phenomenon can be attributed to attenuation of the curing light, differences in monomer reactivity, and a mismatch in the refractive indices of the filler and matrix. However, regarding LinkForce and preheated EverX Flow, although a decrease in HV was observed with increasing depth, the reduction did not reach 80% at 6 mm, indicating that sufficient polymerization depth was still achieved at this thickness. This quantitative finding is further corroborated by the SEM analysis, which revealed a remarkably dense and integrated interface for the preheated EverX Flow and LinkForce for all three regions (Figure 2 and Figure 3). This behavior can be attributed to the dual-cure nature of LinkForce, which allows the material to continue polymerizing chemically in deeper regions where light penetration is limited [53] and the preheating effect for preheated EverX Flow. Consistent with Lempel et al.’s [54] findings, this study demonstrated that preheating significantly enhances the depth of cure for flowable fiber-reinforced composite. Preheating EverX Flow to 55 °C has been substantially shown to increase the degree of conversion, even in deep cavities [48]. However, non-heated EverX Flow fell below the 80% threshold at 6 mm (top surface: 121.00 ± 10.76, 6 mm: 93.65 ± 8.61 HV) with SEM evaluation revealing voids in the middle region and incomplete polymerization in the apical region (Figure 4), indicating that, without preheating, the material failed to achieve a homogenous polymer network in light-restricted areas (Figure 4).

Previous studies have reported that preheating the composite up to 60 °C can increase monomer cross-linking and reactivity, thereby enhancing the depth of cure [55,56]. Accordingly, resin luting materials in this study, except the dual-cure resin–cement, were preheated to 55 °C, which is considered ideal for improving their working properties [57,58]. Preheated G-aenial Injectable maintained the 80% hardness criterion up to 4 mm (top surface: 92.82 ± 6.50, 4 mm: 74.00 ± 6.01), whereas non-heated G-aenial Injectable dropped below the 80% threshold at 3 mm (top surface: 89.77 ± 4.84, 3 mm: 74.52 ± 3.54 HV). Consistent with these findings, SEM micrographs of the preheated G-aenial Injectable revealed a relatively dense structure with air bubbles predominantly localized in the apical region (Figure 5). In contrast, non-heated G-aenial Injectable exhibited significant void formation and structural discontinuities extending to both the middle and apical regions (Figure 6), visually confirming insufficient polymerization at deeper regions and poor material adaptation [59]. Furthermore, the total lack of adequate polymerization at the 6 mm depth in G-aenial Injectable underscores the detrimental impact of light attenuation on the curing efficiency in deep radicular spaces. The beneficial effect of preheating enhanced the depth of polymerization by improving light transmission and polymerization efficiency for both EverX Flow and G-aenial Injectable. However, EverX Flow showed statistically higher HV at each evaluated depth as the fiberglass increases resin’s translucency and light conduction [60]. On the other hand, The G-aenial Injectable composite does not contain reinforcing fibers that lead to directing, scattering, and sustaining the curing light within the material. The absence of a fiber network significantly reduced the depth of cure, as confirmed by HV measurements, which showed almost no polymerization at 6 mm. This insufficient polymerization limits monomer conversion in the apical region of the post space, where light attenuation is already a major challenge. Consequently, the resin matrix remains under-polymerized in deep areas, leading to reduced mechanical stability, higher susceptibility to polymerization shrinkage stresses, and weaker adhesion to intraradicular dentin.

Various methods have been used to evaluate the bond strength between posts and intraradicular dentin. Although conventional tensile tests can be performed on root dentin [61], PBS test is generally the preferred option as it better simulates clinical conditions [62]. Moreover, push-out tests have been shown to offer superior reliability compared to microtensile assessments [43]. In this study, PBS was the preferred approach for evaluating the bonding between preheated and non-heated fiber-reinforced flowable and injectable composites and intraradicular dentin. Regarding polymerization depth and curing efficiency of resin-based materials, microhardness tests are considered a gold-standard approach [63]. The ratio of bottom-to-top microhardness values (B/T) is commonly used to assess polymerization at a given depth, with a B/T ratio of 80% or higher indicating sufficient curing for clinical use [64,65]. A strong correlation was found between the degree of monomer conversion and the Vickers hardness (HV) test [49,66]. It is widely recognized that the microhardness value of a cured resin matrix effectively reflects the degree of conversion, as the mechanical properties of a polymer are inherently dependent on the cross-linking density and the quality of the network formed during polymerization [67]. While the ISO scraping method is known to overestimate the depth of cure [65], a microhardness evaluation was used in the present study to assess the depth of cure. This approach provides a reliable assessment of the materials’ polymerization efficiency, which is also critical for minimizing the presence of unreacted monomers and their potential cytotoxic effects on periapical tissues. By achieving higher HV values through preheating, the stability of the polymer network is enhanced, potentially reducing the leaching of toxic monomers. Therefore, in the present study, microhardness evaluation was used to assess the depth of cure.

Despite the significant findings, the present study has several limitations that should be addressed. Primarily, the absence of thermocycling or any other artificial aging protocol implies that the long-term stability of the adhesion and the fatigue resistance of the preheated resin luting materials remain uncertain. In the clinical environment, restorations are continuously exposed to thermal and mechanical stresses, which can affect their interfacial integrity over time. Moreover, this evaluation was limited to a specific range of fiber-reinforced and injectable composites. The selection of these materials from a single manufacturer was driven by the study’s focus on short-fiber-reinforced technology, which is unique to these products. Since variations in filler morphology, monomer chemistry, and fiber orientation among different manufacturers can significantly influence a material’s response to preheating, these findings should not be generalized to all composites. Furthermore, it is important to note that this in vitro evaluation did not account for clinical challenges such as moisture control or surface contamination, which could interfere with the bonding efficacy of preheated resins in a real-world environment. Additionally, polymerization quality was assessed indirectly using Vickers Microhardness (HV) testing rather than directly via spectroscopic methods such as FTIR. Although HV is a validated method that correlates strongly with the degree of conversion, it does not directly measure the specific double-bond conversion. Furthermore, it should be acknowledged that HV measurements were conducted in standardized molds, which may not fully replicate the complex geometry and light attenuation characteristics of a natural root canal. Consequently, the absence of direct spectroscopic analysis limits a definitive assessment of residual monomers and their potential cytotoxic implications for periapical tissues. Future research incorporating FTIR analysis alongside cell viability assays would provide a more comprehensive understanding of the biological safety of preheated resin materials. Therefore, further in vitro studies that employ thermomechanical aging are necessary to confirm the initial bonding of preheated resin luting materials performance to intraradicular dentin.

## 5. Conclusions

Within the limitations of this study, it can be concluded that preheating significantly improves bonding of fiber-reinforced flowable composite and its interfacial adaptation to intraradicular dentin and suggest a promising and reliable alternative to dual-cure resin–cement for fiber post luting. The light-scattering properties of the material combined with the benefits of preheating can create a more stable and well-polymerized assembly, particularly in the challenging apical part of the root.

## Figures and Tables

**Table 1:** Materials used in the study.

Materials	Manufacturer	Type	Composition	Lot Number
Linkforce	GC Corporation (Tokyo, Japan)	Dual-cure resin–cement	*Paste 1:* DMA, UDMA, bis-GMA, pigments, initiator. *Paste 2:* DMA, bis-MEPP, Bis-EMA, UDMA, BHT barium borate glass, dibenzoyl peroxide, initiator (38% vol, 63% wt)	2301051
EverX Flow	GC Corporation (Tokyo, Japan)	Fiber-reinforced flowable composite	UDMA, TEGDMA, Bis-EMA, micrometer scale glass fiber filler, barium glass (46% vol, 70% wt)	2501271
G-aenial Universal Injectable	GC Corporation (Tokyo, Japan)	Nano-filled injectable composite	Bis-EMA, UDMA, dimethacrylate co-monomers, silica, barium glass (45% vol, 69% wt)	2410311
G-aenial Achord	GC Corporation (Tokyo, Japan)	Universal nanohybrid composite	Bis-EMA, UDMA, TEGDMA, dimethacrylate co-monomers, silica (16 nm), silanizated barium glass (300 nm), silicate glass (41%vol, 82% wt)	2306051
G-Multi Primer	GC Corporation (Leuven, Belgium)	Ceramic primer	Silane, phosphoric acid ester, Ethyl alcohol DMA, MDTP, MDP	2301051
G-Premio Bond Dual Cure Activator (DCA)	GC Corporation (Tokyo, Japan)	Universal adhesive	4-META, 10-MDP, MDTP, methacrylate acid ester, fine powdered silica, acetone, photo initiators, distilled water	2411284 2212161
Fiber Post	GC Corporation (Tokyo, Japan)	Fiber Post	Prefabricated, cross-linked, glass	73530

**Table 2:** The mean and standard deviations of PBS of different resin luting composite materials in coronal, middle and apical thirds.

PBS	Coronal	Middle	Apical	*p* *
LinkForce	16.68 ± 1.54 ^a,A,C^	11.54 ± 2.71 ^b,A^	7.18 ± 1.07 ^c,A^	0.0001
Non-heated EverX Flow	17.81 ± 1.26 ^a,A^	13.37 ± 2.03 ^b,A,B^	9.77 ± 1.68 ^c,B^	0.0001
Preheated EverX Flow	19.69 ± 1.57 ^a,B^	13.99 ± 1.93 ^b,B^	9.43 ± 1.12 ^c,B^	0.0001
Non-heated G-aenial Injectable	13.81 ± 1.62 ^a,D^	6.06 ± 0.74 ^b,D^	3.6 ± 0.68 ^c,C^	0.0001
Preheated G-aenial Injectable	15.66 ± 0.84 ^a,C^	9.18 ± 0.61 ^b,C^	4.72 ± 0.39 ^c,C^	0.0001
*p* *	0.0001	0.0001	0.0001	

Different lowercase letters represent significant differences among root regions (coronal, middle, and apical) within the same material; uppercase letters represent significant differences between resin luting materials within each region. * Statistically significant difference

**Table 3:** Tukey multiple comparison test for the different resin luting composite materials in different root regions.

Tukey Multiple Comparison	EverX Flow	Linkforce	Heated EverX Flow	Heated G-Aenial Injetable	G-Aenial Injetable
Coronal/Middle	0.0001	0.0001	0.0001	0.0001	0.0001
Coronal/Apical	0.0001	0.0001	0.0001	0.0001	0.0001
Middle/Apical	0.0001	0.0001	0.0001	0.0001	0.0001

**Table 4:** Two-way ANOVA for PBS of different types of resin luting composite materials and root region.

	Type III Sum of Squares	df	Mean Square	F	*p*
Intercept	18,597.01	1	18,597.01	9064.96	0.0001
Composite	834.33	4	208.58	101.67	0.0001
Region	2279.57	2	1139.78	555.58	0.0001
Composite × Region	45.82	8	5.73	2.79	0.007

**Table 5:** The mean and standard deviations of HV in different depths.

Hardness	Top	2 mm	3 mm	4 mm	6 mm	*p* *
Linkforce	117.93 ± 7.85 ^A,a^	112.55 ± 7.05 ^A,b^	107.25 ± 6.34 ^A,c^	100.35 ± 6.84 ^A,d^	96.58 ± 5.63 ^A,e^	0.0001
Non-heated EverX Flow	121.00 ± 10.76 ^A,a^	114.48 ± 13.21 ^A,b^	109.23 ± 11.68 ^A,c^	101.8 ± 9.85 ^A,d^	93.65 ± 8.61 ^A,e^	0.0001
Preheated EverX Flow	160.77 ± 5.22 ^B,a^	155.4 ± 5.24 ^B,b^	148.22 ± 6.99 ^B,c^	140.65 ± 6.02 ^B,d^	132.43 ± 5.10 ^B,e^	0.0001
Non-heated G-aenial Injectable	89.77 ± 4.84 ^C,a^	84.67 ± 4.92 ^C,b^	74.52 ± 3.54 ^C,c^	46.73 ± 3.07 ^D,d^	NA	0.0001
Preheated G-aenial Injectable	92.82 ± 6.50 ^C,a^	89.32 ± 5.51 ^C,b^	81.22 ± 5.19 ^C,c^	74.00 ± 6.01 ^C,d^	39.98 ± 5.29 ^C,e^	0.0001
*p* *	0.0001	0.0001	0.0001	0.0001	0.001	

Different lowercase letters represent significant differences between depths within the same resin luting material; uppercase letters signify significant differences between resin luting materials at the same depth. * Statistically significant difference

**Table 6:** Two-way ANOVA for HV of different types of resin luting composite materials and different depth.

Source	Type III Sum of Squares	df	Mean Square	F	*p*
Depth (mm)	9527.28	3	3175.76	695.34	0.0001
Composite	86,175.34	4	21,5443.84	107.89	0.0001
Depth (mm) × Composite	1970.55	12	164.21	35.96	0.0001

**Table 7:** Pearson correlation between push-out bond strength and microhardness tests.

			Push-Out Bond Strength			
			Coronal	Middle	Apical	Overall Correlation
Microhardness	Top	r	0.756	0.770	0.787	0.439
		*p*	0.0001	0.0001	0.0001	0.0001
	2 mm	r	0.744	0.759	0.774	0.432
		*p*	0.0001	0.0001	0.0001	0.0001
	3 mm	r	0.753	0.771	0.789	0.439
		*p*	0.0001	0.0001	0.0001	0.0001
	4 mm	r	0.785	0.824	0.818	0.461
		*p*	0.0001	0.0001	0.0001	0.0001
	6 mm	r	0.679	0.689	0.745	0.355
		*p*	0.0001	0.0001	0.0001	0.0001

## Data Availability

The original contributions presented in this study are included in the article. Further inquiries can be directed to the corresponding author.

## Abbreviations

The following abbreviations are used in this manuscript:

PBS	push-out bond strength
HV	microhardness
Bis-MEPP	bisphenol A-glycidyl methacrylate-phosphoric acid ester,
BHT	Butylated Hydroxytoluene
DMA	dimethylacrylamide,
UDMA	urethane dimethacrylate,
10-MDP	10-methacryloyloxydecyl dihydrogen phosphate,
4-MET	4-methacryloyloxyethyl trimellitate,
Bis-GMA	bisphenol-A-glycidyldimethacrylate,
10-MDTP	10-methacryloyloxydecyl dihydrogen phosphate,
Bis-EMA	Etoksi bisfenolA-dimetakrilat
NaOCl	sodium hypochlorite
EDTA	Ethylenediaminetetraacetic acid
SEM	scanning electron microscope

## References

[1] Şişmanoğlu S. (2020). Restoration of endodontically treated teeth: A review of direct restorative approach. Aurum J. Health Sci..

[2] Dietschi D., Duc O., Krejci I., Sadan A. (2008). Biomechanical considerations for the restoration of endodontically treated teeth: A systematic review of the literature, Part II (Evaluation of fatigue behavior, interfaces, and in vivo studies). Quintessence Int..

[3] Alshabib A., Abid Althaqafi K., AlMoharib H.S., Mirah M., AlFawaz Y.F., Algamaiah H. (2023). Dental fiber-post systems: An in-depth review of their evolution, current practice and future directions. Bioengineering.

[4] Cagidiaco M.C., Goracci C., Garcia-Godoy F., Ferrari M. (2008). Clinical studies of fiber posts: A literature review. Int. J. Prosthodont..

[5] Lazari P.C., Oliveira R.C.N.d., Anchieta R.B., Almeida E.O.d., Freitas Junior A.C., Kina S., Rocha E.P. (2013). Stress distribution on dentin-cement-post interface varying root canal and glass fiber post diameters. A three-dimensional finite element analysis based on micro-CT data. J. Appl. Oral Sci..

[6] Kramer E., Meyer-Lueckel H., Wolf T., Schwendicke F., Naumann M., Wierichs R. (2019). Success and survival of post-restorations: Six-year results of a prospective observational practice-based clinical study. Int. Endod. J..

[7] Wierichs R., Kramer E., Wolf T., Naumann M., Meyer-Lueckel H. (2019). Longevity of composite build-ups without posts—10-year results of a practice-based study. Clin. Oral Investig..

[8] Suni A.O., Lassila L.V., Tuokko J.K., Garoushi S., Vallittu P.K. (2023). Adhesion of individually formed fiber post adhesively luted with flowable short fiber composite. Biomater. Investig. Dent..

[9] Le Bell-Rönnlöf A.-M., Lassila L.V., Kangasniemi I., Vallittu P.K. (2011). Load-bearing capacity of human incisor restored with various fiber-reinforced composite posts. Dent. Mater..

[10] Maletin A., Knežević M.J., Koprivica D.Đ., Veljović T., Puškar T., Milekić B., Ristić I. (2023). Dental resin-based luting materials. Polymers.

[11] Shadman N., Atai M., Ghavam M., Kermanshah H., Ebrahimi S.F. (2012). Parameters affecting degree of conversion of dual-cure resin cements in the root canal: FTIR analysis. J. Can. Dent. Assoc..

[12] Kim Y., Kim S., Kim K., Kwon T. (2009). Degree of conversion of dual-cured resin cement light-cured through three fibre posts within human root canals: An ex vivo study. Int. Endod. J..

[13] Roberts H.W., Leonard D.L., Vandewalle K.S., Cohen M.E., Charlton D.G. (2004). The effect of a translucent post on resin composite depth of cure. Dent. Mater..

[14] Ortiz-Magdaleno M., Bogarin-Topete E.R., Cerda-Cristerna B.I., Gutiérrez-Sánchez M. (2023). Effect of degree of conversion on the surface properties of polymerized resin cements used for luting glass fiber posts. J. Prosthet. Dent..

[15] Juloski J., Goracci C., Radovic I., Chieffi N., Vichi A., Vulicevic Z.R., Ferrari M. (2013). Post-retentive ability of new flowable resin composites. Am. J. Dent..

[16] Silva C.F., Martins V.M., de Paula Melo A., Martins L.C., Santos-Filho P.C.F. (2021). The use of bulk-fill flow in the customization of glass fiber post. Eur. J. Dent..

[17] da Mota Martins V., Silva C.F., Almeida L.M., de Paula M.S., de Sousa Menezes M., Santos-Filho P.C.F. (2019). Bond strength of glass fiber posts cemented with bulk-fill flowable composite resin. Appl. Adhes. Sci..

[18] Bhopatkar J., Ikhar A., Chandak M., Mankar N., Sedani S. (2022). Composite pre-heating: A novel approach in restorative dentistry. Cureus.

[19] Nakano E.L., de Souza A.C., Boaro L.C., Catalani L.H., Braga R., Gonçalves F. (2020). Polymerization stress and gap formation of self-adhesive, bulk-fill and flowable composite resins. Oper. Dent..

[20] Che S., Awang R.A., Adnan M.B.M., Ma X., Gao X., Ismail N.H. (2024). Restorative Strategies for Posterior Teeth Following Endodontic Treatment. J. Nat. Sci. Biol. Med..

[21] AlDabeeb D.S., Alakeel N.S., Alkhalid T.K. (2023). Endocrowns: Indications, preparation techniques, and material selection. Cureus.

[22] (2019). Dentistry-Polymer-Based Restorative Materials.

[23] Santi M., Lins R., Sahadi B., Soto-Montero J., Martins L. (2022). Comparison of the mechanical properties and push-out bond strength of self-adhesive and conventional resin cements on fiber post cementation. Oper. Dent..

[24] Hajjaj M.S., Alhowirini L.F., Alghamdi R.S., Merdad Y.M., Filemban H.K., Bawazir M., Alothman K.A., Turkestani N.A., Alzahrani S.J. (2025). Effects of Preheating on the Mechanical Properties of Dental Composites. Crystals.

[25] Oskoee P.A., Nooroloyouni A., Azar F.P., Oskoee J.S., Ashraf A.P. (2015). Effect of resin cement pre-heating on the push-out bond strength of fiber post to root canal dentin. J. Dent. Res. Dent. Clin. Dent. Prospect..

[26] Hardan L., Bourgi R., Cuevas-Suarez C.E., Zarow M., Kharouf N., Mancino D., Villares C.F., Skaba D., Lukomska-Szymanska M. (2021). The bond strength and antibacterial activity of the universal dentin bonding system: A systematic review and meta-analysis. Microorganisms.

[27] Faria-e-Silva A.L., Pfeifer C.S. (2020). Development of dual-cured resin cements with long working time, high conversion in absence of light and reduced polymerization stress. Dent. Mater..

[28] Uctasli S., Boz Y., Sungur S., Vallittu P.K., Garoushi S., Lassila L. (2021). Influence of post-core and crown type on the fracture resistance of incisors submitted to quasistatic loading. Polymers.

[29] Lassila L., Oksanen V., Frater M., Vallittu P.K., Garoushi S. (2020). The influence of resin composite with high fiber aspect ratio on fracture resistance of severely damaged bovine incisors. Dent. Mater. J..

[30] Fráter M., Lassila L., Braunitzer G., Vallittu P.K., Garoushi S. (2020). Fracture resistance and marginal gap formation of post-core restorations: Influence of different fiber-reinforced composites. Clin. Oral Investig..

[31] Vieira C., Bachmann L., Chaves C.D.A.L., Silva-Sousa Y.T.C., Da Silva S.R.C., Alfredo E. (2021). Light transmission and bond strength of glass fiber posts submitted to different surface treatments. J. Prosthet. Dent..

[32] Haralur S.B., Alasmari T.A., Alasmari M.H., Hakami H.M. (2022). Light transmission of various aesthetic posts at different depths and its effect on push-out bond strength, microhardness of luting cement. Medicina.

[33] Le Bell-Rönnlöf A.-M., Jaatinen J., Lassila L., Närhi T., Vallittu P. (2019). Transmission of light through fiber-reinforced composite posts. Dent. Mater. J..

[34] Forster A., Sáry T., Braunitzer G., Fráter M. (2017). In vitro fracture resistance of endodontically treated premolar teeth restored with a direct layered fiber-reinforced composite post and core. J. Adhes. Sci. Technol..

[35] Sarkis-Onofre R., Skupien J., Cenci M., Moraes R., Pereira-Cenci T. (2014). The role of resin cement on bond strength of glass-fiber posts luted into root canals: A systematic review and meta-analysis of in vitro studies. Oper. Dent..

[36] Sari C., Bala O., Akgul S., Alp C.K. (2025). Effect of using different materials and restorative techniques on cuspal deflection and microleakage in endodontically treated teeth. BMC Oral Health.

[37] Szczesio-Wlodarczyk A., Garoushi S., Vallittu P., Bociong K., Lassila L. (2024). Polymerization shrinkage of contemporary dental resin composites: Comparison of three measurement methods with correlation analysis. J. Mech. Behav. Biomed. Mater..

[38] Rosado L., Münchow E., de Oliveira E., Lacerda-Santos R., Freitas D., Carlo H., Verner F. (2023). Translucency and Radiopacity of Dental Resin Composites–Is There a Direct Relation?. Oper. Dent..

[39] Prisco D., De Santis R., Mollica F., Ambrosio L., Rengo S., Nicolais L. (2003). Fiber post adhesion to resin luting cements in the restoration of endodontically-treated teeth. Oper. Dent.-Univ. Wash..

[40] Aleisa K., Habib S.R., Ansari A.S., Altayyar R., Alharbi S., Alanazi S.A.S., Alduaiji K.T. (2021). Effect of luting cement film thickness on the pull-out bond strength of endodontic post systems. Polymers.

[41] Keles Z.H., Isik V., Turunc R., Sismanoglu S. (2025). Silane-Containing Self-Adhesive Resin Cement vs. Conventional Strategies in Fiber Post Application: A Push-Out Bond Strength and Failure Mode Study. Appl. Sci..

[42] Rocha A.T., Gonçalves L.M., Vasconcelos A.J.d.C., Matos Maia Filho E., Nunes Carvalho C., De Jesus Tavarez R.R. (2017). Effect of anatomical customization of the fiber post on the bond strength of a self-adhesive resin cement. Int. J. Dent..

[43] Goracci C., Tavares A.U., Fabianelli A., Monticelli F., Raffaelli O., Cardoso P.C., Tay F., Ferrari M. (2004). The adhesion between fiber posts and root canal walls: Comparison between microtensile and push-out bond strength measurements. Eur. J. Oral Sci..

[44] Özcan M., Volpato C.A.M. (2020). Current perspectives on dental adhesion: (3) Adhesion to intraradicular dentin: Concepts and applications. Jpn. Dent. Sci. Rev..

[45] Pinto S.T.P., Machado H.d.P.M., Ramos M.d.C., Gelio M.B., Oliveira C.R.d.M., Kuga M.C., Tanomaru-Filho M., Reis J.M.d.S.N. (2025). Influence of different cementation protocols on the bond strength of glass fiber posts to root dentin: An in vitro study with quantitative and qualitative analyses. Int. J. Adhes. Adhes..

[46] Shimizu S., Sawada T., Asano A., Kan T., Noda M., Takemoto S. (2021). Effects of different bonding systems with various polymerization modes and root canal region on the bond strength of core build-up resin composite. J. Prosthodont. Res..

[47] Hagino R., Mine A., Aoki-Matsumoto M., Miura J., Yumitate M., Ban S., Ishida M., Takaishi M., Van Meerbeek B., Yatani H. (2024). Effect of filler contents on the bond strength of CAD/CAM resin crowns: New resin primer versus conventional silane agents. J. Prosthodont. Res..

[48] Srikumar G., Barde S.P., Gajpal A., Kumari R., Phafat A., Patel P. (2025). An In vitro Comparative Evaluation of the Effect of Preheating of EverX Posterior, Bulk Fill and G-aenial Posterior Composite Resins on Their Microhardness and Depth of Cure. Adv. Hum. Biol..

[49] Degirmenci A., Can D.B. (2022). Pre-heating effect on the microhardness and depth of cure of bulk-fill composite resins. Odovtos-Int. J. Dent. Sci..

[50] Ayub K.V., Santos G.C., Rizkalla A.S., Bohay R., Pegoraro L.F., Rubo J.H., Santos M. (2014). Effect of preheating on microhardness and viscosity of 4 resin composites. J. Can. Dent. Assoc..

[51] Basheer R.R., Hasanain F.A., Abuelenain D.A. (2024). Evaluating flexure properties, hardness, roughness and microleakage of high-strength injectable dental composite: An in vitro study. BMC Oral Health.

[52] Rueggeberg F., Caughman W.F., Curtis J. (1994). Effect of light intensity and exposure duration on cure of resin composite. Oper. Dent..

[53] Windle C.B., Hill A.E., Tantbirojn D., Versluis A. (2022). Dual-cure dental composites: Can light curing interfere with conversion?. J. Mech. Behav. Biomed. Mater..

[54] Lempel E., Őri Z., Szalma J., Lovász B.V., Kiss A., Tóth Á., Kunsági-Máté S. (2019). Effect of exposure time and pre-heating on the conversion degree of conventional, bulk-fill, fiber reinforced and polyacid-modified resin composites. Dent. Mater..

[55] Sajjan G.S., Dutta G.S., Varma K.M., Satish R.K., Pulidindi A.K., Kolla V.B. (2022). One-year clinical evaluation of bulk-fill composite resin restorations plasticized by preheating and ultrasonics: A randomized clinical trial. J. Conserv. Dent. Endod..

[56] Fróes-Salgado N.R., Silva L.M., Kawano Y., Francci C., Reis A., Loguercio A.D. (2010). Composite pre-heating: Effects on marginal adaptation, degree of conversion and mechanical properties. Dent. Mater..

[57] Kincses D., Böddi K., Őri Z., Lovász B.V., Jeges S., Szalma J., Kunsági-Máté S., Lempel E. (2021). Pre-heating effect on monomer elution and degree of conversion of contemporary and thermoviscous bulk-fill resin-based dental composites. Polymers.

[58] Lousan do Nascimento Poubel D., Ghanem Zanon A.E., Franco Almeida J.C., Vicente Melo de Lucas Rezende L., Pimentel Garcia F.C. (2022). Composite resin preheating techniques for cementation of indirect restorations. Int. J. Biomater..

[59] Albelasy E.H., Raghip A.G., Ismail H.S. (2025). Internal adaptation and micromorphological analysis of a new self-cure resin composite. J. Clin. Exp. Dent..

[60] Garoushi S., Vallittu P.K., Lassila L.V. (2008). Depth of cure and surface microhardness of experimental short fiber-reinforced composite. Acta Odontol. Scand..

[61] Nikaido T., Takano Y., Sasafuchi Y., Burrow M., Tagami J. (1999). Bond strengths to endodontically-treated teeth. Am. J. Dent..

[62] Le Bell A.-M., Tanner J., Lassila L.V., Kangasniemi I., Vallittu P.K. (2004). Bonding of composite resin luting cement to fiber-reinforced composite root canal posts. J. Adhes. Dent..

[63] Saati K., Khansari S., Mahdisiar F., Valizadeh S. (2022). Evaluation of microhardness of two bulk-fill composite resins compared to a conventional composite resin on surface and in different depths. J. Dent..

[64] Rodriguez A., Yaman P., Dennison J., Garcia D. (2017). Effect of light-curing exposure time, shade, and thickness on the depth of cure of bulk fill composites. Oper. Dent..

[65] Flury S., Hayoz S., Peutzfeldt A., Hüsler J., Lussi A. (2012). Depth of cure of resin composites: Is the ISO 4049 method suitable for bulk fill materials?. Dent. Mater..

[66] Skrinjaric T., Gorseta K., Bagaric J., Bucevic Sojcic P., Stojanovic J., Marks L.A. (2025). Comparison of microhardness and depth of cure of six Bulk-Fill resin composites. J. Compos. Sci..

[67] Shimura R., Nikaido T., Yamauti M., Ikeda M., Tagami J. (2005). Influence of curing method and storage condition on microhardness of dual-cure resin cements. Dent. Mater. J..

